# Enterocolitis

**Published:** 1906-06

**Authors:** O. W. Cobb

**Affiliations:** Easthampton, Mass.


### ENTEROCOLITIS.

BY O. W. COBB, M.D.,

EASTHAMPTON, MASS.

I was called last August to see an eight-months'-old boy who was said to be dying of cholera infantum. He had been treated by two capable men, both of whom agreed that the child could not possibly outlive the day. Every conventional remedy had been tried and the favorite methods of both men had been exhausted. They frankly admitted that all had been done that could be done. I found the patient almost moribund and displaying all the symptoms of a child dying of what I diagnosed as enterocolitis. The symptoms, to my mind, were classic, despite the previous diagnosis. The case was turned over to me at 8 a.m., August 7. A trained nurse was already on this case. She is an unusually competent woman, in whom I have the most implicit confidence. Then began one of the hardest battles of some years in my practice. I ordered high enemas of Glyco-Thymoline in 25% solution and warm. Used four ounces at a time with a soft rubber catheter once every three hours. The child could retain nothing, was in frightful pain and passing constantly thin foul smelling discharge tinged with blood. The child was emaciated to the last degree, and for several days before I was called had been in a semi-conscious state. The poor little baby was a pitiful sight. For nourishment I ordered several combinations to be administered, an ounce at a time, as a rectal clyster following the enemas of Glyco-Thymoline.

I know it is not good practice to give hypodermics to an infant, but this was a grave case. My predecessor had ordered gr. 1-64 morphine, gr. 1-960 atropi, sub. q. every four hours if needed, with strychnine 1-240 gr. if necessary. I continued this, as the baby was often in intense pain, and these seemed to be no other way. This was my plan of campaign, and I am both thankful and pleased that it was successful. The baby improved from the first, but so slowly that it was scarcely discernible to the parents, but the nurse and myself saw it. After three days the child could take some nourishment per oram. I then gave 2 m. Glyco-Thymoline in one ounce of water every two hours before feeding. It began to have short periods of natural rest and the discharges were in every way improved. At the end of a week, August 14th, the improvement was quite marked, but we did not relax our vigilance. The hypodermics, except of strychnine, were discontinued. The enemas were continued fifteen days, once every three hours, then at less frequent intervals for a month, then once a day for six weeks. The recovery of the little patient was long and slow, but uneventful. The mother and nurse were devoted and ably seconded my efforts. At this time the baby is a strong, rosy youngster.

It gives me great pleasure to tell you of this case. The experience may be of value, and it certainly proves to my satisfaction at least, the potential possibilities of Glyco-Thymoline in gastro-intestinal work. May you be speeded in your good work.

